# Gaucher Disease in Bone: From Pathophysiology to Practice

**DOI:** 10.1002/jbmr.3734

**Published:** 2019-06-24

**Authors:** Derralynn Hughes, Peter Mikosch, Nadia Belmatoug, Francesca Carubbi, TimothyM Cox, Ozlem Goker‐Alpan, Andreas Kindmark, PramodK Mistry, Ludger Poll, Neal Weinreb, Patrick Deegan

**Affiliations:** ^1^ Royal Free London NHS Foundation Trust and University College London UK; ^2^ Department of Internal Medicine 2 Landesklinikum Mistelbach, Austria, and Medical University Vienna, Externe Lehre Vienna Austria; ^3^ Referral Center for Lysosomal Diseases, Department of Internal Medicine, University Hospital Paris Nord Val de Seine, Assistance Publique‐Hôpitaux de Paris Clichy France; ^4^ Department of Biomedical Metabolic and Neural Sciences, University of Modena and Reggio Emilia, NOCSAE Hospital, AOU Modena Italy; ^5^ Department of Medicine University of Cambridge Cambridge UK; ^6^ Lysosomal Disorders Research and Treatment Unit Fairfax VA USA; ^7^ Department of Endocrinology and Diabetology Uppsala University Hospital Uppsala Sweden; ^8^ Department of Internal Medicine (Digestive Diseases) Yale University School of Medicine New Haven CT USA; ^9^ Practice of Radiology and Nuclear Medicine Duisburg‐Moers, Heinrich‐Heine University Düsseldorf Duisburg Germany; ^10^ Departments of Human Genetics and Medicine (Hematology) Miller School of Medicine, University of Miami FL USA; ^11^ Lysosomal Disorders Unit, Addenbrooke's Hospital Cambridge UK

**Keywords:** BIOMARKERS, BONE DISEASE, GAUCHER DISEASE, OSTEONECROSIS, OSTEOPOROSIS, RADIOLOGY, THERAPEUTICS

## Abstract

Gaucher disease (GD) is a rare, genetic lysosomal disorder leading to lipid accumulation and dysfunction in multiple organs. Involvement of the skeleton is one of the most prevalent aspects of GD and a major cause of pain, disability, and reduced quality of life. Uniform recommendations for contemporary evaluation and management are needed. To develop practical clinical recommendations, an international group of experienced physicians conducted a comprehensive review of 20 years’ of the literature, defining terms according to pathophysiological understanding and pointing out best practice and unmet needs related to the skeletal features of this disorder. Abnormalities of bone modeling, reduced bone density, bone infarction, and plasma cell dyscrasias accompany the displacement of healthy adipocytes in adult marrow. Exposure to excess bioactive glycosphingolipids appears to affect hematopoiesis and the balance of osteoblast and osteoclast numbers and activity. Imbalance between bone formation and breakdown induces disordered trabecular and cortical bone modeling, cortical bone thinning, fragility fractures, and osteolytic lesions. Regular assessment of bone mineral density, marrow infiltration, the axial skeleton and searching for potential malignancy are recommended. MRI is valuable for monitoring skeletal involvement: It provides semiquantitative assessment of marrow infiltration and the degree of bone infarction. When MRI is not available, monitoring of painful acute bone crises and osteonecrosis by plain X‐ray has limited value. In adult patients, we recommend DXA of the lumbar spine and left and right hips, with careful protocols designed to exclude focal disease; serial follow‐up should be done using the same standardized instrument. Skeletal health may be improved by common measures, including adequate calcium and vitamin D and management of pain and orthopedic complications. Prompt initiation of specific therapy for GD is crucial to optimizing outcomes and preventing irreversible skeletal complications. Investing in safe, clinically useful, and better predictive methods for determining bone integrity and fracture risk remains a need. © 2019 The Authors. *Journal of Bone and Mineral Research* Published by Wiley Periodicals Inc.

## Introduction

### Pathophysiology, diagnosis, and treatment of Gaucher disease

Gaucher disease (GD) is a lysosomal disorder leading to lipid accumulation. The lysosome is an intracellular organelle that degrades and recycles biological macromolecules derived either endogenously, from digestion of cellular components (autophagy), or from the breakdown of material incorporated from outside the cell by phagocytosis. Sphingolipids, amphiphilic compounds with a lipophilic moiety based on the amino‐alcohol sphingosine, are found in all plasma membranes. They mediate diverse cellular functions and serve as specific receptors and cell‐recognition markers. Ultimately, they are delivered to the lysosomal compartment during the course of membrane turnover in endocytosis and phagocytosis. Glucosylceramide is the sphingolipid degraded by the acid hydrolase glucocerebrosidase (also named acid β‐glucosidase), whose role it is to cleave the final sugar, a glucose moiety, from the ceramide component. Glucosylsphingosine, the deacylated form of the glucosylceramide, is water‐soluble and hence freely diffusible and may be relevant in the pathogenesis of GD.

About one in 5000 live‐born infants has a lysosomal disorder, with GD and Fabry disease (both sphingolipidoses) probably the most frequent. GD is caused by the inherited deficiency of the acid hydrolase glucocerebrosidase causing an accumulation of its substrate glucosylceramide. Glucosylceramide accumulates mainly in the macrophages, transforming them into Gaucher cells.

GD was once regarded as the most frequent lysosomal disease; however, Fabry disease has ultimately proved more frequent. The birth incidence is calculated to be approximately 1/100,000, depending on the ethnic composition of the population. The inheritance pattern is autosomal recessive. More than 250 mutations responsible for the enzymatic deficiency have been identified in the human glucocerebrosidase gene (chromosome 1q22), and approximate genotype–phenotype correlations can be applied.^1^

A clinical subclassification has been applied that is based on age of onset and the presence and severity of neurological involvement. Infantile GD (type 2) is rare and causes death in the first 2 years of life: There is neuronopathic disease with bulbar palsy, opisthotonus, and minor visceral enlargement. It is invariably fatal and does not respond to either systemic or intrathecal enzyme replacement therapy (ERT). Neurological disease in children, adolescents, and young adults is termed subacute neuronopathic (type 3). In such patients, neurological disease is less severe and associated with supranuclear gaze palsies, ataxia, a central defect of auditory processing, myoclonus, and occasionally seizures. Subacute neuronopathic disease is not always fatal and often improves with bone marrow (BM) transplantation and ERT (nonneurological aspects only).^2^, ^3^

The most frequent form of GD, in Europe and the Americas, is the so‐called adult nonneuronopathic form (type 1). This disease is found in all populations, but is overrepresented in Jews of Ashkenazi origin, in whom a single mutation (p.Asn409Ser, formerly called N370S) dominates. The enzyme deficiency in type 1 disease is typically incomplete, with up to 20% of normal activity remaining. Although the condition does not commonly affect the nervous system, visceral and skeletal manifestations are prominent. Extrapyramidal disease resembling Parkinson disease develops in midlife in approximately 5% of patients.^4^, ^5^

The pathognomonic abnormality of GD is the presence of storage cells, which are activated macrophages (Gaucher cells). The Gaucher cells are found in the splenic sinusoids, replace the Kupffer cells of the liver and alveolar macrophages of the lung, and infiltrate the BM. Of all cell types, the macrophages carry the greatest storage burden. The measurable remaining enzyme activity is sufficient to catabolize the endogenous turnover of glucosylceramide in other cells types, including neurons (in type 1 disease), but is insufficient to cope with the greater flux of the sphingolipid substrate in phagocytosing cells exposed to the additional load associated with the lipid fraction of membranes of engulfed cells. As a consequence, storage macrophages undergo phenotypic changes that include not only enlargement, but also alteration in cell‐surface receptor expression, inflammatory cytokine and chemokine production, and the secretion of enzymes, including proteases, into the extracellular space. The immune phenotype of the characteristic Gaucher cell is that of an “alternatively activated” macrophage, usually associated with chronic inflammation and scarring when seen in other disorders such as scleroderma and sarcoidosis. As a result of tissue macrophage distribution, splenomegaly is almost universal, with associated hypersplenism and thrombocytopenia. Anemia is less common, but when severe, is associated with BM failure caused by the infiltration and replacement of hematopoietic marrow. Pulmonary infiltration and lymphadenopathy are infrequent manifestations, usually in children with severe disease. Bone manifestations are the subject of this review and are discussed in detail below.

GD is accompanied by many plasma and metabolic derangements. These can include, in decreasing order of frequency, a polyclonal increase in immunoglobulins, monoclonal gammopathy, multiple myeloma, and B‐cell lymphoma—conditions that are important causes of death in adult patients with type 1 GD; their cause is unknown. Low‐density lipoprotein and high‐density lipoprotein cholesterol fractions are abnormal in the plasma. The basal metabolic rate is increased. Some lysosomal enzymes are elevated in the plasma, including angiotensin‐converting enzyme, tartrate‐resistant acid phosphatase, and hexosaminidase, members of the cathepsin family of proteases, and a human chitinase, chitotriosidase. Chitotriosidase has proved to be very useful for monitoring GD activity in response to treatment and may reflect the severity of the disease. The concentration of this enzyme is sometimes elevated by a factor of several hundred above normal in untreated GD.

The mean age of death in a single large series was 60 years^6^, ^7^ during the pretreatment era, but this longevity does not take into account the poor quality of life of most affected individuals. Some patients homozygous for “mild” missense mutations in the glucocerebrosidase gene may escape detection and remain asymptomatic throughout a long adult life.

The diagnosis of GD is based on glucocerebrosidase activity measured in leukocyte preparations, whereas spleen tissue, liver biopsy material, or BM aspirates may show the characteristic, generally oligonucleate, storage cells demonstrating striated cytoplasm on Leishman staining or pink sheets in tissue sections stained with H&E. Molecular analysis may identify mutant glucocerebrosidase alleles that cause this disease and may assist in the diagnosis and investigation of family members at risk of this recessive disorder.

Before the advent of ERT, treatment for GD was palliative and supportive: This remains the case in much of the developing world. It has been estimated that only 10% of potential patients worldwide are receiving specific treatment.^8^ BM transplantation has been undertaken in a few infants and children with rapidly progressive disease, including those with the subacute neuronopathic form, type 3. BM transplantation is no longer in routine use because of the accompanying risks of the procedure and constraints in the supply of donors.

Patterns of medical care clearly vary throughout healthcare systems worldwide. Patients are most often under the principal care of hematologists, metabolic specialists, geneticists, pediatricians, gastroenterologists, or rheumatologists. Supportive care is also provided by orthopedic surgeons, neurologists, and other specialists.

The first ERT, alglucerase (Ceredase; Sanofi Genzyme, Cambridge, MA, USA), was introduced in 1991 in the form of the physiological enzyme extracted from human placenta and modified to reveal terminal mannose residues. A recombinant form, imiglucerase (Cerezyme; Sanofi Genzyme), that is similarly modified for effective macrophage targeting became available in 1997. After a few months of enzyme administration, most patients show an improvement in the blood parameters of disease activity: The platelet count rises, with a reduction in hepatosplenomegaly and an improvement in the severe fatigue that accompanies GD. Quality‐of‐life measures also show clear improvement in clinical studies. Similar effects are noted with the use of the more recently licensed enzyme therapies velaglucerase (VPRIV; Shire Human Genetic Therapies, Lexington, MA, USA) and taliglucerase (Elelyso; Protalix Biotherapeutics, Karmiel, Israel). A detailed comparison of specific treatment options is beyond the scope of this review.

At present, there is no agreed protocol for therapy in adults with GD. However, the application of simple defined therapeutic goals with close monitoring of individual patients has much to recommend it. Achievement of key goals and amelioration of disease‐associated parameters is more rapid when the recommended dose of enzyme therapy (approximately 60 U/kg every other week) is administered. ERT, although very expensive (as much as €258,000/US$288,000 per year),^9^ is a successful treatment for GD. Hypersensitivity and immune reactions are very rare, and many patients in different countries receive therapy at home.

In response to the inconvenience of parenteral administration, there have been initiatives to develop alternative treatments, including the use of oral agents that inhibit the formation of the substrate delivered to macrophages. The first such agent approved, miglustat (Zavesca; Actelion Pharmaceuticals, San Francisco, CA, USA), reduced the sphingolipid content of circulating white cells and had modest effects on laboratory and clinical parameters of GD activity. Miglustat is licensed in the United States and Europe for use in patients with mild‐to‐moderate type 1 GD for whom ERT is unsuitable. The occurrence of peripheral neuropathy after long‐term administration in a few patients and short‐lived unwanted side‐effects, including diarrhea, may restrict the indications for its use. Additional orally active compounds are now authorized: The substrate‐reducing agent and ceramide analogue eliglustat (Cerdelga, developed by Sanofi Genzyme) has shown beneficial effects similar to those of enzyme therapy and has demonstrated good efficacy against aspects of bone disease in clinical trials.

### Bone disease in GD patients

Skeletal manifestations are prevalent at all ages and are the principal cause of pain, disability, and reduced quality of life.^10^, ^11^, ^12^ However, there remains a need for uniform recommendations for bone disease evaluation and management.^12^, ^13^ Physicians, including pediatricians, may be unfamiliar with bone pathophysiology and the complexity of the skeletal manifestations arising throughout life. There is a need to enhance awareness and to improve diagnosis, characterization, quantification, and treatment of skeletal and BM pathology in patients with GD.

GD affects the BM and the mineralized components of bone. Changes include (1) BM infiltration and plasma cell dyscrasias; (2) modeling and remodeling abnormalities of bones, resulting in developmental changes and loss of bone mineral (osteopenia/osteoporosis), cortical thinning, lytic lesions, and fragility fractures; and (3) osteonecrosis and related phenomena (medullary infarctions, osteosclerosis, cortical infarcts, and joint destruction and deformities).

Histopathological examination reveals not only Gaucher cells, but also osteolytic lesions, necrosis, fibrosis, loss of trabecular bone, and failure of hematopoiesis. Bioactive sphingolipids induce a cascade of changes that affect bone vascularity, intramedullary pressure, the immune environment, hematopoiesis, and the function of cells that reside in bone, ultimately disturbing skeletal growth and bone modeling and remodeling.^14^ Several of these effects (eg, marrow infiltration by pathological macrophages and osteopenia) are modified by specific therapies, but the underlying pathophysiology of bone disease remains poorly understood. Clearly, secondary changes (complications, not universally present, but which ensue in some patients—eg, osteonecrosis) and tertiary changes (chronic manifestations of the secondary changes, such as osteoarthritis, fracture deformity, and joint replacement) are not reversed by specific disease‐modifying treatment.^14^

Of 2004 patients enrolled in the International Collaborative Gaucher Group (ICGG) Gaucher Registry from 1991 to 2000, between 76% and 94% of those with GD type 1 (GD1) had a radiological manifestation of bone disease, including marrow infiltration, Erlenmeyer flask deformity, or osteonecrosis.^15^ Bone disease is often asymptomatic^15^ and may sometimes progress despite apparently effective disease‐modifying treatment.^16^, ^17^

### Objectives

To develop useful guidance in the assessment and management of bone manifestations of GD for the practicing clinician, we—an international group of physicians collectively having treated over 1500 patients and having published over 150 articles on GD and bone complications of GD—met in Amsterdam, Netherlands, in May 2016 (a meeting financially supported by Sanofi Genzyme). In the preparatory phase of the meeting, a comprehensive review of the literature was done by a broad search using Embase and PubMed (January 1995 to December 2015) for published articles with the terms “Gaucher disease” and “bone” diseases, including related terms and synonyms, which resulted in 521 articles. International congress abstracts indexed in Embase from 2014 to April 2016 were also included. All abstracts were prescreened for original and clinical data (resulting in 290 remaining references) and reviewed by topic groups of two or three experts and focusing on four major topics: BMD, osteonecrosis, BM disease, and other bone publications. Each topic group reviewed the articles for relevance of information and quality of evidence to support statements on natural history, monitoring, and treatment recommendations. At the face‐to‐face meeting in Amsterdam, the morning was used to present and complete all key findings and outcomes of the four topic groups to the whole group and to discuss perspectives and interpretations. The afternoon was used to complete missing data and to discuss and vote on best recommendations. We found that the division into four topics was somewhat artificial and overlapping; therefore, we decided to organize best practices and unmet needs in GD‐related bone disease into three key skeletal compartments: (1) *the bone marrow* (Gaucher cell infiltration, macrophage function, osteoclast differentiation, interactions among bone and marrow or immune cells, soluble messengers and biomarkers, fibrosis, and extraosseous “Gaucheromas”); (2) *trabecular and cortical bone modeling and remodeling defects*, delayed bone growth, and bone mineral loss and their consequences; and (3) *medullary and cortical bone infarction* (osteonecrosis, osteosclerosis, acute and chronic infection, and bone and joint deformity). The structure of the discussion and the division into three compartments was driven by an attempt to link elements of bone involvement according to putative pathogenic mechanisms rather than morphology, and to clarify and standardize the terminology of GD bone involvement. In most areas, consensus was reached by weighing arguments, clinical experience, and the available literature to set out practical recommendations. When it was clear that agreement could not be achieved, we identified aspects of GD‐related bone disease that are not fully characterized or remain controversial.

## The Bone Marrow

In patients with GD, organs containing mononuclear phagocytes, including the BM, liver, and spleen, are infiltrated by enlarged, glycosphingolipid‐laden macrophages called Gaucher cells.^18^ Gaucher cells are described as having an eccentric nucleus and a striated (or “crumpled silk”) cytoplasm,^19^ but atypical variants (eg, multinucleated forms, erythrophagocytotic cells, cells with foamy cytoplasm) exist in untreated patients and may confound the diagnosis.^20^ However, Gaucher cells are not pathognomonic of GD (“pseudo‐Gaucher” cells are sometimes found in the marrow of patients with myeloma and acute leukemias, for example), and the diagnosis is dependent on the demonstration of deficient glucocerebrosidase activity in leukocytes, cultured skin fibroblasts, or dried blood spots.^21^

Gaucher cells progressively and centrifugally displace the normal, triglyceride‐rich adipocytes from the adult marrow,^22^ initially in the axial skeleton and finally in the extremities.^18^ This replacement occurs in the opposite direction to the physiological replacement of red, hematopoietic marrow by the fatty marrow as part of normal development—thus complicating evaluation in young people. The infiltrative process is established early in the course of the disease and usually before bone symptoms develop.^23^

### Pathogenesis

Although individuals with extensive infiltration of the marrow by Gaucher cells are more likely to suffer bone complications, it remains unclear how the infiltration affects BM organization and function.^18^, ^24^

In human cell‐culture studies, erythropoiesis, myeloid proliferation and differentiation, and mesenchymal stem cell development are impaired by inhibition of glucocerebrosidase before pathological glucosylceramide storage is detected and morphological changes occur.^25^ Hematopoiesis may be downregulated by various bioactive glycosphingolipids whose intracellular levels and relative proportions are abnormal in GD.^25^ Hematopoiesis and skeletal remodeling may also be affected by the displacement of normal BM adipocytes by Gaucher cells.^22^, ^26^ BM mesenchymal stromal cells (which give rise to the bone‐forming osteoblasts, the osteocytes, and bone‐lining cells, as well as BM adipocytes) have been reported to have an altered cytokine‐ and prostaglandin‐expression profile (the inflammatory secretome). These changes may promote not only reduced BMD, by increasing osteoclast numbers and activity, but also proliferation and activity of plasma cells, and thus the generation of polyclonal and monoclonal gammopathies.^27^

GD is associated with an increased risk of cancer in general (relative risk 1.7 compared with the general population) and BM‐based hematological malignancies (estimated risk 3.5 to 12.7) in particular, specifically multiple myeloma (estimated risk 25.0 to 51.1).^28^, ^29^ The clonal immunoglobulin in GD patients binds to glucosylsphingosine,^28^, ^29^, ^30^ concentrations of which are greatly increased in GD patients as a direct consequence of glucocerebrosidase deficiency.^31^

### Histological and imaging findings

The histological appearance of infiltrated BM is heterogeneous, and pathological examination of the femoral head reveals areas of vital bone adjacent to Gaucher‐cell‐infiltrated marrow, areas of nonspecific chronic inflammation and fibrosis, islands of necrosis in osteocyte‐free areas of avital bone, and areas of normal hematopoietic marrow.^32^ Nevertheless, it remains unclear whether the severity of bone disease is correlated with the extent of Gaucher‐cell infiltration^33^, ^34^ or their particular features.

In adults, marrow infiltration by Gaucher cells can be seen by MRI, in which T1‐weighted spin‐echo sequences show areas of reduced signal intensity known as dark marrow, reflecting greater water content. The amount and distribution of dark marrow seen by MRI reflect the extent of infiltration of the BM by Gaucher cells (containing water) as they displace the fat in the marrow compartment.^18^, ^24^ The pattern of infiltration provides information about disease severity: an inhomogeneous (type B) pattern has been described to reflect greater irreversibility of disease than a homogeneous type A pattern.^24^, ^35^ The extent of BM infiltration can be evaluated by quantitative chemical shift imaging (QCSI),^36^ which allows measurement of the fat fraction of BM in the vertebrae. A fat fraction less than 0.23 is associated with bone complications.^24^ However, QCSI is complex, expensive, and of very limited availability.^24^

In common practice, the extent of BM infiltration by Gaucher cells is evaluated semiquantitatively from MRI data of the spine, pelvis, and lower limbs by using one of several scoring systems. The systems most widely used in clinical practice include the Bone Marrow Burden (BMB) score, which correlates well with fat fraction measured by QCSI,^36^ the Düsseldorf Gaucher Score (DGS),^35^, ^37^ and the vertebra‐to‐disk ratio (VDR).^38^ The BMB score, DGS, and VDR have shown utility for the radiological follow‐up of BM infiltration in multiple studies.^39^ Other bone disease scoring systems are used less frequently.^40^, ^41^, ^42^, ^43^, ^44^, ^45^ An estimation of disease severity based on hematological and visceral manifestations, as well as bone manifestations, is provided by the GD1 disease‐severity scoring system (GD1‐DS3), in which 42% of the total score is determined by bone manifestations (hematological manifestations provide 32% and visceral manifestations 26% of the total score).^46^ The Italian Gaussi‐I severity scoring system proposed a clinically useful categorization of the various BM infiltration scores.^47^

MRI‐based scoring of marrow infiltration is not as reliable in children and young adults as in mature adults. Values of the BMB score, DGS, and VDR do not accurately reflect clinical status in younger patients because of their higher proportion of hematopoietic (rich in water and poor in fat) marrow.^39^ However, the VDR, which changes with age and fluctuates according to the water content of the intervertebral disk, is more reliable in younger patients because they lack the disk degeneration seen in older patients.^39^ In children, vertebral BM infiltration can also be evaluated by measuring the apparent diffusion coefficient, a parameter that correlates inversely with several markers of GD severity.^48^

BM infiltration may also be reliably represented by scintigraphy using the lipophilic cationic agent ^99m^Tc‐sestamibi,^49^ and a normalized score has been proposed that takes into account the values from several MRI‐based scoring systems and the scintigraphic score.^50^, ^51^ Scintigraphy using ^99m^Tc‐sestamibi provides an alternative method by which to evaluate marrow infiltration in children, but is not widely available and involves radiation exposure.

The replacement of marrow fat by GD‐related tissue may be exaggerated in the presence of massive splenomegaly with anaemia: Appreciable red cell pooling because of hypersplenism stimulates erythropoietin release and an element of erythroid expansion.^18^

### Treatment and response evaluation

Approved treatments available to date for GD1 are the ERTs imiglucerase, velaglucerase alfa, and taliglucerase alfa (the last not approved in the EU) and the substrate**‐**reduction therapies (SRTs), eliglustat and miglustat. These have been reviewed in detail elsewhere.^11^ Realizing that relevant differences between the various molecules may exist, we would like to emphasize that for ease of reading, here we have used the umbrella term, ERT. Most experience has been gathered with imiglucerase^52^; we therefore recommend reviewing the cited references and the local label when considering a particular treatment for potential benefits in bone biology and clinical efficacy.

Bone complications respond to ERT more slowly than visceral and hematological manifestations of GD.^53^ The BMB score has been shown to improve by 2 or more points in patients receiving ERT^54^ and appears to stabilize after 5 years of therapy,^55^ reaching near‐normal values after 7 years of ERT. However, an increase in the fat fraction measured by QCSI has been reported to occur within a year of ERT.^56^ In children receiving ERT, most clinical parameters (including BMD) have been reported to normalize or near‐normalize within 8 years.^57^ In treatment‐naïve adults in clinical trials of eliglustat, mean BMB score improved by 2 points after 18 months of eliglustat therapy,^58^ and mean LS *T*‐scores moved from the osteopenic range to the normal range after 2 years; improvement continued for up to 8 years.^59^ Gaucher cell infiltration in the femur, as shown by the amount of dark marrow, has been shown to decrease or remain stable with eliglustat.^59^ In adults with GD1 who switched to eliglustat after having been stabilized on long‐term ERT (mean of 10 years), a repeated measures, mixed‐model analysis showed that the least‐square mean LS *Z*‐score, which was in the normal reference range at baseline, increased by 0.29 (*P* < 0.001) after 4 years of eliglustat therapy.^60^

### Our recommendations

#### Diagnosis of BM infiltration

Initial and serial measurement of blood counts is standard practice for evaluating disease severity and response to treatment. Thrombocytopenia is mainly attributable to hypersplenism, as shown by near‐invariable normalization after splenectomy, whereas BM dysfunction is more relevant to anemia. Chitotriosidase can be a valuable marker for serial assessment of GD patients,^23^, ^61^, ^62^, ^63^ but activity is related to the total burden of storage, not specifically bone pathology. In patients genetically deficient in chitotriosidase activity, the chemokine CCL18/PARC can be used to monitor therapeutic response. Serum glucosylsphingosine shows promise as a reliable and specific biomarker for both diagnosis and monitoring of treatment response in GD.^64^, ^65^, ^66^

A BM biopsy is not necessary for diagnosis or monitoring of GD, but may be appropriate in a patient in whom hematological malignancy is suspected. Assessment of serum immunoglobulins, serum‐free light chains, and urinary Bence‐Jones proteins may suggest a diagnosis of myeloma. Diagnosis of myeloma by microscopic cytological examination can be hindered by large numbers of Gaucher cells. Plasma cells may be atypical; identification of intracytoplasmic immunoglobulin may help to distinguish plasma cells from Gaucher cells.

MRI can provide semiquantitative assessment of marrow infiltration, an insight into the degree of reversibility of the marrow infiltration. An inhomogeneous pattern of marrow infiltration is associated with the presence of bone infarction.^67^

#### Monitoring and scoring

Our recommendations for monitoring the BM are shown in Table 1. MRI is the gold standard for monitoring bone involvement in GD patients, taking into account the patient's age. Below 19 years, we suggest including tibiae (BM in tibiae is converted to fatty marrow around age 9 years). Below age 9, it is very difficult to assess red marrow infiltration. Although plain radiography is not as sensitive or precise as MRI or DXA, it can provide useful and clinically meaningful information in locations where MRI and DXA are not available. In this instance, children should be assessed annually and the radiographs evaluated by an orthopedist or radiologist experienced in GD.^68^ The utility of whole‐body MRI,^24^ proton MR spectroscopy,^69^ and QCSI is theoretical because the availability of these modalities is limited. Data on the use of CT‐PET and MRI‐PET in GD are limited.

**Table 1:** The Expert Panel's Recommendations for Monitoring Bone Involvement in Gaucher Patients

Area to assess	Technique	Parameter to measure or scoring system	Comments
**BMD (osteopenia, osteoporosis)**			
Adults: LS and left and right hips In clinical practice, usually only one hip is scanned, but a dual hip scan is recommended to provide a comparison In patients with LS collapse or hip osteonecrosis, use a less‐affected site for measurement (e.g. distal third of the radius and/or calcaneus of the non‐dominant leg)	DXA Serial DXA scans should be performed using the same device When assessing therapeutic effects or serial evaluations, changes need to be related to the smallest significant changes detectable by the centre and machine used Patients aged <50 years (including premenopausal women): evaluate Z‐score Check once every 2–4 years Patients aged >50 years, postmenopausal women (evaluate T‐score), and glucocorticoid‐treated patients Check every 1–2 years Assess every 24–36 months (untreated patients) or every 36 months (patients whose goals for skeletal involvement have been achieved on ERT^a^) Repeat at shorter intervals (e.g. 12–24 months) if rapid loss of bone mass is likely (e.g. patients on corticosteroid therapy) During treatment, allow at least 12 months between BMD evaluations	Essential parameters to measure: Bone mineral content (g) Bone area (cm^2^) BMD (g/cm^2^) Calculated Z‐score Z‐score <‐2.0 indicates reduced BMD An increase in BMD Z‐score with treatment is usually accompanied by improvement in bone pain and bone crises Postmenopausal women and men older than 50 years Use the T‐score Interpret using the WHO classification of osteopenia and osteoporosis	Relatively inexpensive, widely available, and associated with low radiation exposure, but It may give erroneous results in areas of damaged bone – may result in false high or false low BMD depending whether lytic or infarction lesions are present – may result in false high BMD if compression fractures have occurred A DXA‐based BMD does not correlate with the risk of fracture in all patients (e.g. premenopausal women) Reimbursement differs by location – e.g. in the USA, most insurance plans and Medicare will pay for DXA only biannually unless a clear medical need can be demonstrated Interpretation of scans can be aided by plain radiography Consider radiation exposure
Children: total body less head	DXA in children older than 5 years	Z‐score, age‐adjusted BMD	Particularly consider radiation exposure
**Active bone disease**			
Pelvis, vertebrae, and limbs	MRI	BM oedema Bone infarcts Periosteal bleeding	Active bone disease is indicated by a high signal on STIR sequences
	T1, STIR, orientation depending on the anatomical site		
Whole body	MRI	DGS, BMD, VDR	Significant limitations make these options impractical for widespread use Expense, complexity, low availability
	Whole body: coronal T1, STIR		
	Axial skeleton: sagittal T1, T2, STIR, composed of cervical, thoracic, and LS		
	Abdomen and pelvis: axial T2, T2‐fat‐sat		
BM fat fraction	QCSI, STIR‐weighted sequences	Fat fraction in LS	Correlates with fracture risk Limited availability
**Marrow infiltration and bone involvement (pathological fractures, osteonecrosis, lytic lesions, Erlenmeyer flask deformity)**			
BM cavity	MRI	Semi‐quantitative scoring Dusseldorf‐Gaucher Score (DGS) Bone‐marrow Burden (BMB) score	Gold standard for assessment of bone involvement in Gaucher patients
Axial skeleton (BM fraction) and lower extremities	Spin‐echo technique, T1, T2, STIR,		
	LS: sagittal, T1, T2, STIR,		
			More accurate than plain radiography for detecting early skeletal involvement
	lower extremities: coronal, T1, T2, STIR		
			Can provide undistorted images of marrow cavity
Axial and appendicular skeleton e.g. fractures, osteoarthrosis, osteosyntheses	Plain radiography (depending of the anatomical site, e.g. hips, knee, LS, etc.)	Radiomorphological imaging	Can be used for diagnosis and for specific lesions Widely available Provides important baseline information on skeletal status
	
			Not suitable for monitoring acute bone crises
			Consider radiation exposure, especially in children
	Aspiration biopsy should not be used for diagnosis of GD		Biopsy should be performed only when malignancy or other haematological disease is suspected
**Malignancy**			
BM	Biopsy may be indicated	Karyotype to detect chromosomal abnormalities consistent with multiple myeloma Mutation analysis may also be sometimes indicated to detect multiple clones	Treatment should follow guidelines appropriate for the tumour type Take care to minimize myelotoxic effects of chemotherapy
Blood	Serum protein electrophoresis and immunofixation test at baseline and follow‐up Every 2 years (patients aged 40–50 years) Annually (patients aged >50 years) MGUS screening is not necessary in children or young adults	Identify abnormal Ig profile (including free light chains in serum and urine)	Multiple myeloma does not preclude use of ERT^a^ Aggressive myeloma may not allow sufficient time for ERT^a^ to take effect

^a^ ERT is used in this article as an umbrella term and includes various molecules used in ERT and SRT. Relevant differences may exist and review of the local label of a particular treatment is recommended when considering potential benefits in bone biology and clinical efficacy.

#### Treatment

Whether the treatment of choice is ERT or SRT, prompt initiation is key to optimizing outcomes.^14^, ^70^

For physicians who are relatively inexperienced in treating GD, we encourage multidisciplinary collaboration to facilitate clinical decision‐making; the team should include a radiologist experienced in GD.

In case of a malignancy, treatment should follow guidelines appropriate for the tumor type, taking into account the complexities of GD, and care should be taken to optimize BM function in relation to the delivery of myelotoxic chemotherapy.^71^ The presence of GD in itself should not, however, influence the decision to treat the malignancy.

### Remaining unmet needs

Specific biochemical markers of Gaucher cell‐related bone disease are needed to allow frequent noninvasive monitoring of bone pathology and its response to therapy.^14^

Although understanding of the pathophysiology of GD‐related malignancy has recently advanced, further work is required to facilitate the targeted intervention and prevention of this disease manifestation.

## Trabecular and Cortical Bone: Modeling and Remodeling Defects

This category of GD‐related bone disease was created to bring together those features linked by the putative common pathological mechanism of imbalance between bone formation and breakdown, between the actions of osteoblasts and osteoclasts. Here we include a disorder of primary bone modeling that takes place as part of long bone growth: the Erlenmeyer flask deformity. We also include the spectrum of diffuse low BMD found in patients with GD as measured by DXA, cortical bone thinning, and consequential fragility fractures and focal osteolytic lesions.

### Modeling and remodeling defects

Long bones include the diaphysis (the hollow shaft), flared metaphyses below the growth plates, and the epiphyses above the growth plates. With the cessation of endochondral ossification at the growth plates and consequent fusion of the metaphyses and epiphyses at the end of adolescence, longitudinal bone growth ends. Children with severely symptomatic GD often have retarded growth as well as delayed puberty. Although bone growth may accelerate with the eventual onset of puberty, some children continue to have short stature as adults. When ERT is begun before growth plate closure, most children and adolescents with GD reach their expected midparental height.^57^, ^72^, ^73^

Bones also undergo radial growth that is determined by a net excess of new periosteal osteoblastic bone formation over endosteal osteoclast‐mediated bone resorption.

To accommodate biomechanical stresses, long bones typically undergo an osteoclast‐driven modeling process and change in shape and contour. This process also primarily occurs during childhood and adolescence.^74^

### Erlenmeyer flask deformity

**Figure 1:**
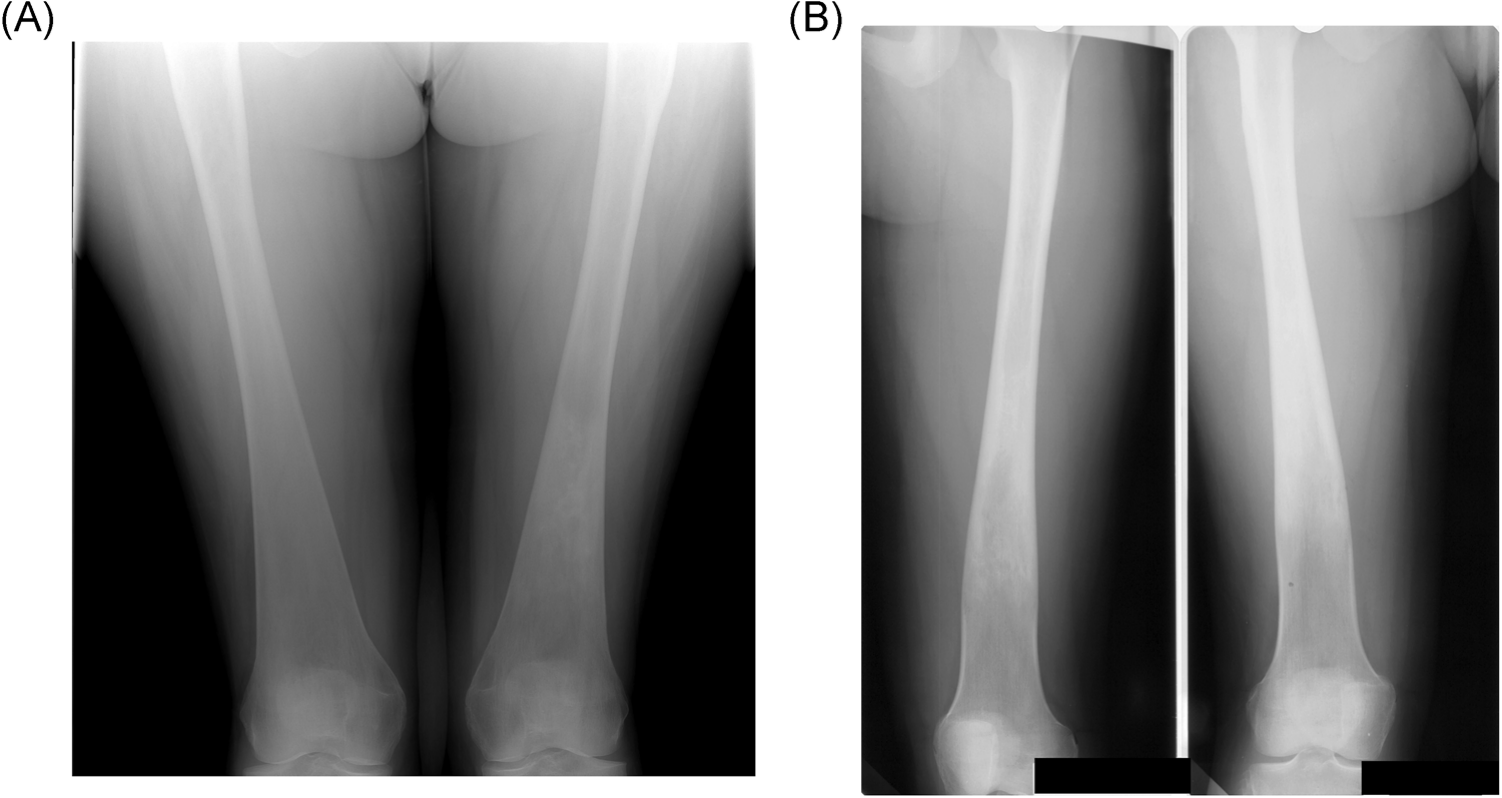
Erlenmeyer flask deformity. (*A*) Typical appearance. Radiograph of the lower femora, showing the triangular outline of the metaphysis. Note the indistinct boundary between the cortex and medulla, typical of the Erlenmeyer flask deformity in Gaucher disease (GD). Incidentally, there is an area of serpiginous sclerosis in the left femoral metaphysis, suggestive of osteonecrosis. (*B*) Atypical appearance. Radiograph of the lower femora of a woman with GD who began enzyme replacement therapy at the age of 12 years. The proximal metaphysis has features similar to those in *A*, but the distal metaphysis has the more normal, trumpet‐shaped outline of the distal femur, with a clear border between the cortex and medulla. We speculate that the normal modeling process of endochondral ossification took place from the time of initiation of therapy

One outcome of defective modeling based on BM infiltration in GD is the Erlenmeyer flask deformity, which starts before puberty.^11^, ^14^ The flask‐like appearance (Fig. 1) results from enlargement of the metaphyseal area and consequent absence of the typical concave dimetaphyseal curve.^11^, ^14^ The deformity, generally asymptomatic, can be seen in many of the tubular or long bones of the skeleton, occasionally even the phalanges, in GD patients, but it does not affect bones that arise in membranes (eg, the skull vault). Although it is reported in up to 80% of adults with GD1, principally at the lower femur, and may provide a diagnostic clue, the Erlenmeyer flask deformity is not unique to GD, and its significance is uncertain.^11^, ^12^, ^14^ Definition and quantification of the abnormality have been attempted.^75^

The sign is also observed in other bone diseases^76^, ^77^, ^78^, ^79^, ^80^ in which osteoclast function is thought to be impaired. It is present in a mouse model deficient in tartrate‐resistant acid phosphatase, an enzyme essential for the resorptive function of osteoclasts.^81^, ^82^, ^83^

The clinical significance of the Erlenmeyer flask deformity is unclear. To date, it has not been found to be associated with fragility fracture or osteonecrosis.^84^

### Osteopenia and osteoporosis

#### The bone turnover cycle

Throughout life, bones are constantly remodeled to maintain mineral homeostasis and preserve bone strength by repairing stress microfractures. Osteocytes, about 90% of adult skeleton cells, function as a mechanosensor and send signals to both osteoclasts and osteoblasts to remodel or maintain bone mass. The remodeling process involves tight coupling of osteoblast–osteoclast function and is regulated by the ratio of RANKL to osteoprotegerin (OPG): High OPG levels protect against excessive osteoclastic bone resorption.^85^ Sclerostin (an inhibitor of osteoblastic activity), prostaglandin E2 (a stimulator of osteoblast activity), cytokines (IL‐1, IL‐6), the protease cathepsin K, parathyroid hormones, estrogen, vitamin D [1,25(OH)_2_D_3_], and osteocalcin are also well‐known players in bone turnover, which determines bone mass and strength. The β‐catenin signaling pathway plays an essential role in bone growth and development. Targeted deletion of β‐catenin in osteocytes results in a fragile, porous skeleton highly susceptible to fracture.^86^

An excess of osteoclastic bone resorption over osteoblastic bone formation leads to net bone mineral loss. Imbalanced bone remodeling and bone mineral loss characteristically occur in postmenopausal women and, to a lesser extent, in ageing men and sedentary people.^74^

#### Experimental evidence in GD

Experimental studies provide support for the role of aberrant osteoblastic and osteoclastic activity in the development of osteopenia and osteoporosis in GD. Other cells, including mesenchymal stem cells, may also be adversely affected by the lipid accumulation that characterizes GD.^87^

In tissue culture, glucocerebrosidase‐deficient peripheral blood mononuclear cells (PBMCs) differentiate into osteoclasts more rapidly and more vigorously than do monocytes with normal glucocerebrosidase activity, and the functional bone resorptive capacity of glucocerebrosidase‐deficient osteoclasts in vitro is enhanced relative to their normal counterparts.^88^ Also, in ex vivo cell cultures and in vivo experiments in irradiated mice, glucocerebrosidase‐deficient mesenchymal stromal cells (nonhematopoietic osteoblast precursors in the BM) isolated from 10 patients with GD1 had impaired ability to proliferate and differentiate into osteoblasts.^88^, ^89^

#### Markers of osteoblast and osteoclast activity in GD patients

In the healthy population, markers of bone turnover are highly expressed in childhood and especially during pubertal growth acceleration. These osteoblast and osteoclast activity markers decline after completion of growth.

The significance of bone turnover markers in GD has been explored, but findings so far reported are not conclusive, possibly because of diverse experimental conditions and the failure of several studies to take account of the effects of age, pubertal development, and treatment status. In one well‐conducted case‐control study of children and adolescents with untreated GD, serum concentrations of osteocalcin (a marker of osteoblastic bone formation) and type 1 collagen C‐terminal telopeptide (a marker of osteoclastic bone resorption) were lower than those in controls and as low during childhood and adolescence as in adulthood.^90^

Serum glucosylsphingosine may be a pathologically relevant biomarker as it has been shown to mediate osteoblast dysfunction in GD.^87^

Cathepsin K has been identified as the principal expressed protein of the osteoclast.^27^ Cathepsin K is highly active in the cleavage of the bone matrix proteins collagen type 1 and osteonectin, and its role in bone resorption, modeling, and turnover has been clearly demonstrated.^91^ Enhanced cathepsin K expression has been observed in patients with GD.^92^

This perturbation of bone remodeling in GD1 may result in a failure to achieve a peak bone mass equivalent to that of normal adults.^93^ Whether there is a mechanistic link between pubertal delay in GD (discussed in the Modeling and remodeling defects section) and the suppression of bone turnover needs to be established.

### Other factors affecting bone health

There is a general appreciation that vitamin D concentrations are low in GD. Mikosch and colleagues measured concentrations of 25‐OH vitamin D in 74 GD patients in the UK at several points throughout the year.^94^ They found a high prevalence of low vitamin D concentrations, according to a range of cut‐off points. Vitamin D concentrations correlated weakly with bone density measurements, with some seasonal and inverse correlation between vitamin D and parathyroid hormone, indicating that, at least in the winter months, vitamin D deficiency was of physiological importance.

#### Measurement of bone mineral mass

Low bone mineral mass can be detected by bone densitometry (ie, DXA) as a reduction in BMD.^15^ DXA should be performed at the LS (L1 to L4) and hip (total hip or femoral neck) in adults and lumbar + (total body‐minus‐head) in children, considering methodological precision and least significant change detectable by individual equipment. The minimum age for densitometric examinations is 5 years. The parameters obtained from DXA measurements are bone mineral content (BMC) in grams, area in cm^2^, and BMD in g/cm^2^. DXA should be interpreted using the *Z*‐score (difference between the individual's measured value of BMD and the normal population mean) as a reference value in younger individuals.^95^, ^96^ *Z*‐scores below ‐2 indicate reduced BMD.^97^ In postmenopausal women and in men older than 50 years, BMD is expressed in terms of the *T*‐score (calculated in relation to young adults of the same sex)^84^ and is interpreted using the WHO classification of osteopenia and osteoporosis, which defines osteopenia as a *T*‐score between ‐1 and ‐2.5^98^ and osteoporosis as *T*‐scores less than ‐2.5. In children, the total‐body‐minus‐head scan is mandatory; measurement of femoral BMD is not reliable because of the high variability in skeletal maturity, bone size, and pubertal stage.^99^

#### Bone mineral assessment and risk of fracture in GD

An analysis of data from the ICGG Gaucher Registry has shown that a low BMD of the LS is associated with a high risk of fracture of the spine or femur in GD1 patients.^10^ ICGG Gaucher Registry data also indicate that 55% of all registry patients have investigator‐defined osteopenia.^100^ In one study, fragility fractures were reported in 28% (cumulative incidence) of 100 adult GD patients of median age 49 years (range 19 to 85 years).^84^ This figure may be compared with an 18% cumulative incidence of fracture (presumably traumatic and fragility fractures combined) in a separately published, large control group of a similar age and sex distribution.^101^ It is possible that ICGG Registry data represent an underestimate of the fracture prevalence in GD patients because asymptomatic vertebral fractures may not have been recorded.

A *Z*‐score of ‐1 or less in GD patients increases the risk of a fragility (low trauma) fracture by a factor of 5,^12^, ^70^ which is a significantly higher estimate than that commonly associated with osteoporotic fracture in population studies.^102^

A BMD below the expected range for chronological age (*Z*‐score < ‐2.0) occurs early in patients with GD1 and may become apparent by the age of 5 years.^52^

In adults with GD the average lumbar BMD *Z*‐score is approximately ‐1.0, but with a wide distribution.^52^ Patients who have undergone splenectomy have a lower bone density than nonsplenectomized patients,^95^ but it is not clear whether this is simply a reflection of greater disease severity or an effect of splenectomy.

**Figure 2:**
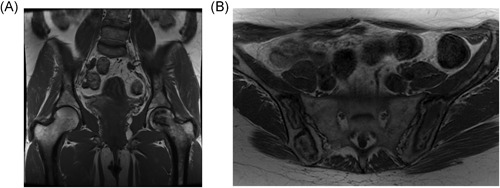
Osteonecrosis. (*A*) Typical. T1‐weighted MR image of the pelvis, showing a geographic area of low signal in the head of the left femur. Note that the joint surface remains intact in this case; therefore, there is no deformity or degenerative change in the hip joint. (*B*) Atypical. T1‐weighted MR image of the pelvis in a different patient showing diffuse low geographic area of low and high signal through the pelvic bones bilaterally. The radiologic changes in (*B*) occurred gradually over years, on enzyme therapy, and without the typical symptoms of bone crisis

**Figure 3:**
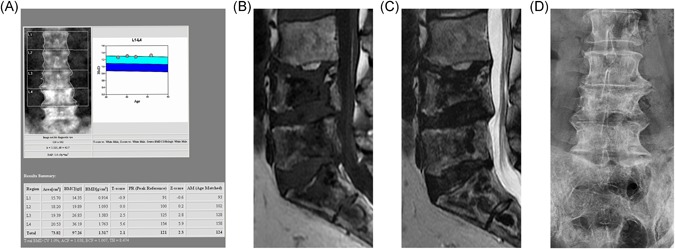
The lumbar spine of a patient with GD and recurrent bone crises despite therapy. All images taken several years after last acute bone crisis. (*A*) DXA image. Note the increased BMD in L3 and L4. These vertebrae were not excluded from the report, despite the variation in BMD among vertebrae, contrary to best practice. The total lumbar vertebral BMD reported is therefore artifactually elevated. (*B*) T1‐weighted MR image showing widespread, low‐signal geographic changes in L4, L5, and the sacrum, features indicative of osteonecrosis. Central endplate depression is also seen. (*C*) T2‐weighted image showing double‐line sign indicative of osteonecrosis best seen in S1 and S2 segments and areas of low signal in the bodies of L4 and L4. Low signal in both T1‐ and T2‐weighted images indicates osteosclerosis. (*D*) Plain anteroposterior lumbar spine radiograph in the same patient show osteosclerosis of the body of L4. The increased density at L4 and L5 is therefore a result of osteosclerosis, consequent upon osteonecrosis, although a contribution from endplate depression cannot be excluded

Assessment of bone mass by DXA in GD may be complicated by focal anatomical changes and focal disease. Osteonecrosis of the femoral head (Fig. 2A) with subsequent sclerotic and arthritic changes makes assessment of that site by DXA inappropriate, and compressed fractured vertebrae need to be excluded. Changes in the water and fat composition of the marrow may also affect the measured BMD, as certain assumptions are made regarding the marrow fat content that may not be true in GD. What may be less obvious to DXA practitioners who are unaccustomed to GD is that osteonecrosis can take place within the vertebral bodies and may result in sclerosis (Fig. 3 *A‐D*), and that relatively small areas of bone within the field of view of DXA scans can undergo necrosis and sclerosis, giving rise to a step‐change increase in BMD. Other confounding factors in DXA measurements in adults include the presence of osteophytes, vascular calcifications, and calculi, which can lead to overestimation of bone mass. It should also be recalled that the DXA score is only one of many determinants of fracture risk that include such elements as smoking status, alcohol consumption, physical activities, use of concurrent medications, and vitamin D deficiency.

#### Osteolytic lesions

**Figure 4:**
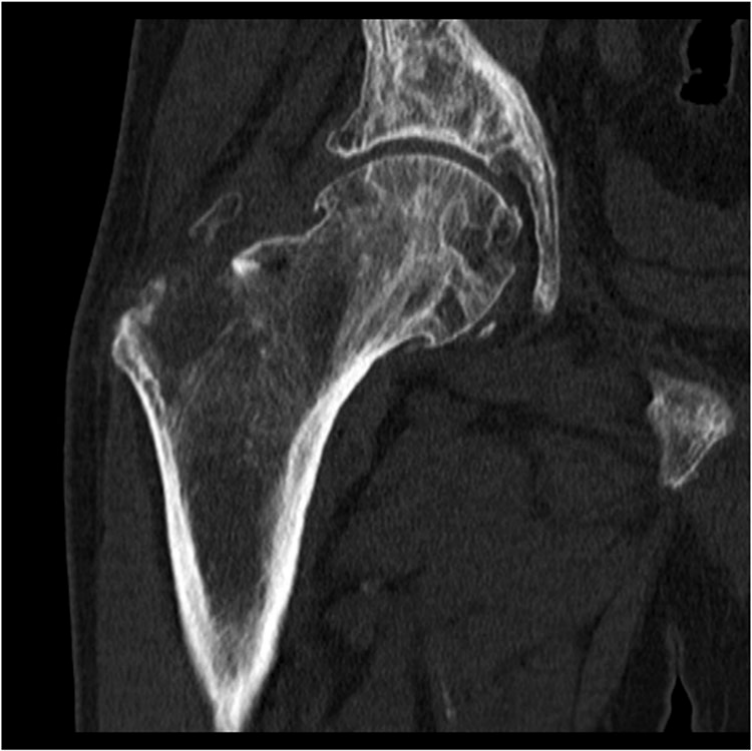
CT image of the right hip, showing a lytic and expanding lesion (Gaucheroma) of the greater trochanter, superiorly displacing fragments of bone

**Figure 5:**
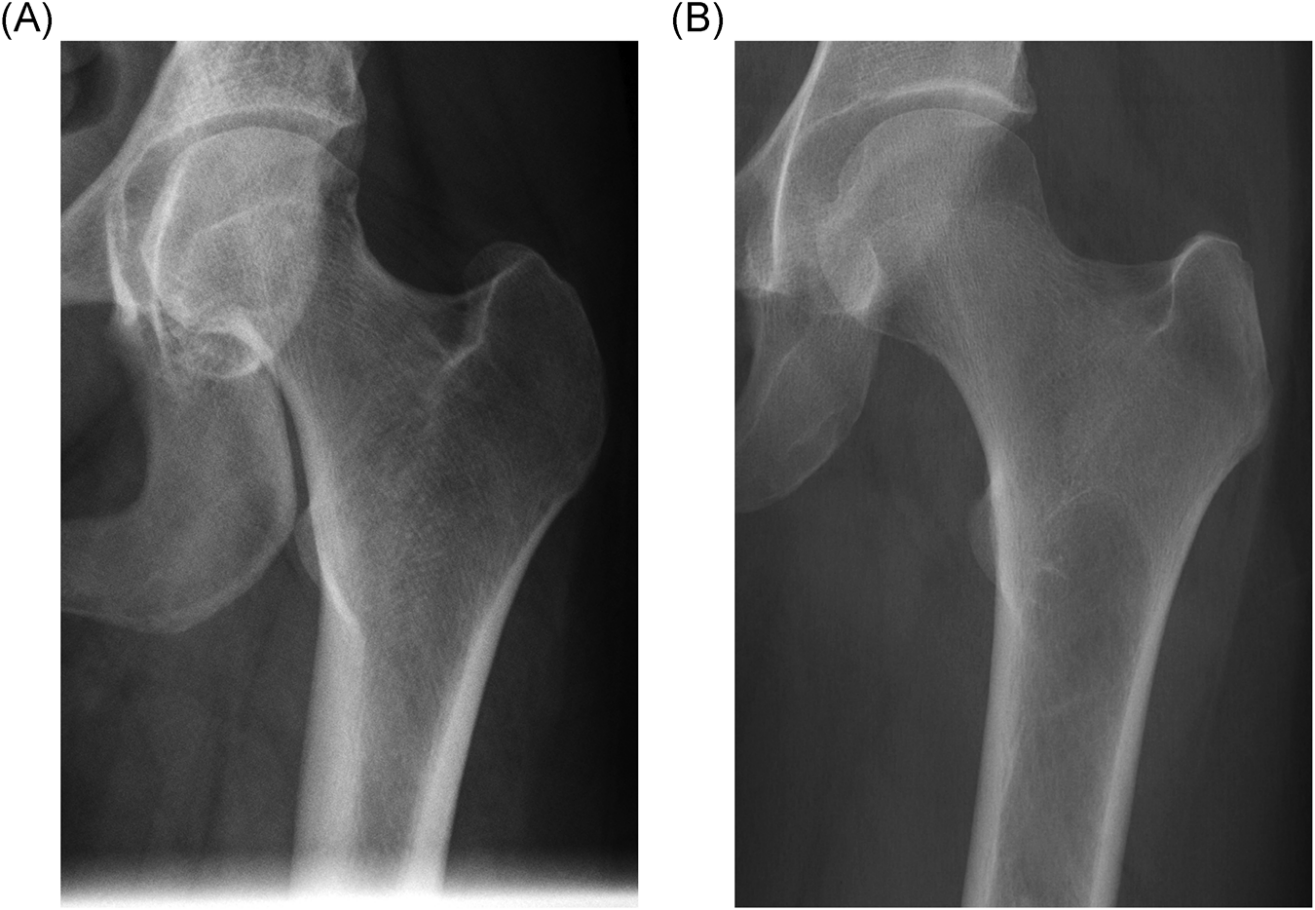
Plain radiographs of the proximal left femora of two patients with Gaucher disease. (*A*) Normal cortical thickness in the femoral shaft of the left proximal femur. (*B*) Reduced cortical thickness in the femoral shaft of the left proximal femur. Note the thinner cortex in comparison with *A*

Focal osteolytic lesions (also called lytic lesions; Fig. 4) are uncommon in GD1 patients.^84^, ^100^, ^103^ They are thought to be caused by the action of cysteine proteases secreted by Gaucher and related cells^27^ in areas where the intermedullary space is tightly packed with infiltrated macrophages.^11^ They have a typically “worm‐eaten” appearance because of a rarefied cortex and dentate endosteum; the cortical thinning (Fig. 5 *A, B*) is an important risk factor for fractures.^11^, ^12^

It is important to differentiate GD‐related osteolytic lesions from those occurring in malignancies (eg, multiple myeloma, plasmacytoma).^11^

#### Kyphosis and gibbus deformity

**Figure 6:**
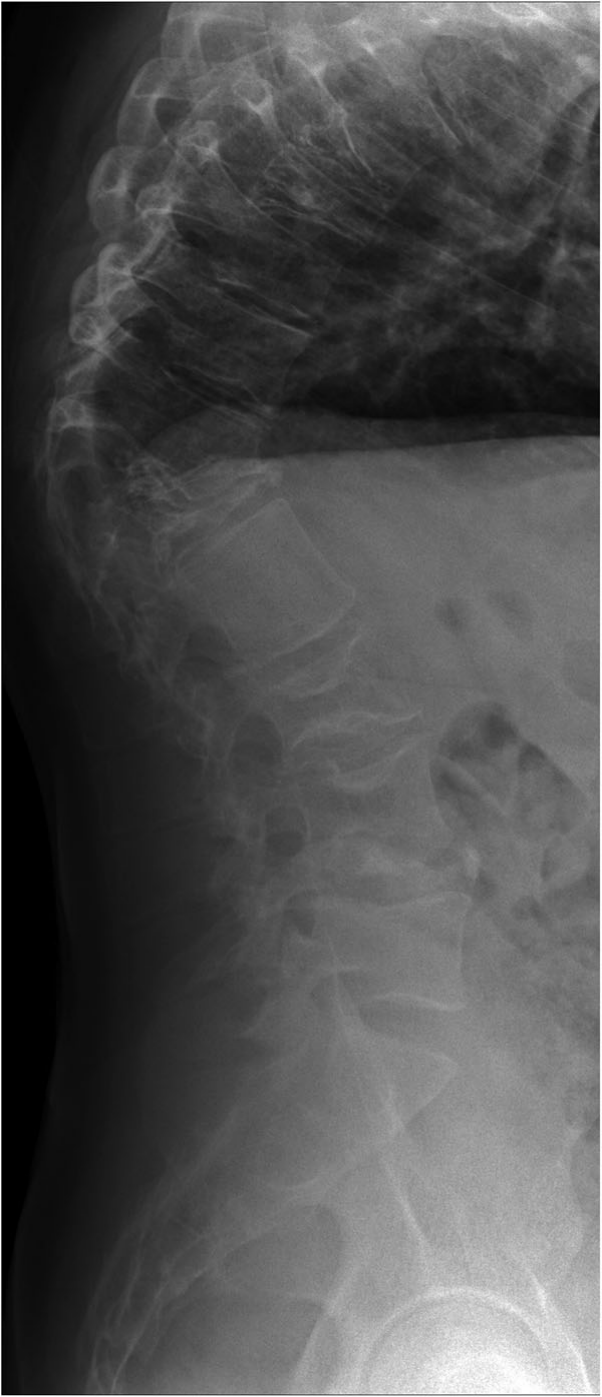
Lateral radiograph of the spine of a patient with type 3 GD, showing an abruptly angulated thoracic kyphosis

Thoracic kyphosis may develop in any patient with GD as a result of vertebral fragility fractures resulting from low bone mass. There is, however, a distinct entity, gibbus deformity found in many patients with chronic, neuronopathic (type 3) GD (especially those homozygous for L444P) that manifests as a sharply angulated deformity of the spine, usually at the thoracolumbar junction (Fig. 6). This deformity usually develops in late childhood or early adolescence and can give rise to severe height loss, chest wall deformity, and restricted breathing. Many patients undergo corrective surgery using metal Harrington rods and spinal fusion. The cause of the gibbus deformity is debated and unclear, but it does not correlate with other skeletal manifestations of GD and is apparently not preventable with ERT.

### Our recommendations

#### Monitoring and scoring

In asymptomatic adult patients, we recommend careful DXA of the LS and left and right hips, considering potentially confounding focal disease (see Table 1). For children, total‐body‐minus‐head DXA is required. Serial DXA evaluations should consider the *Z*‐score (or *T*‐score), BMD, and BMC.

When considering the relation between GD and fracture, it is important to exclude the influence of comorbidities that increase the fracture risk (eg, multiple myeloma, steroid use).

We recommend not using QCT of the LS routinely to avoid unnecessary radiation exposure, although it may occasionally be helpful in resolving questionable DXA results and to assess critical cortical‐thinning associated with lytic lesions. We also recommend not using ultrasonography for the assessment of bone density and quality.

#### Treatment

In general, optimal skeletal health should be ensured by following common clinical practice, including adequate calcium and vitamin D therapy according to local guidelines. Improvement in osteopenia has been widely reported in GD patients treated with ERT or with SRT in observational studies and in clinical trials. ERT improves bone mass in all age groups: The greatest effect is seen in younger subjects during the period when peak BMD is accrued.^52^ Early and sustained treatment is crucial, as a clear dose–response relation has been demonstrated.^53^ So far, no evidence has emerged for an effect of specific Gaucher treatment on the risk of fracture. A single double‐blind, placebo‐controlled clinical trial has demonstrated the efficacy of oral bisphosphonate therapy in improving BMD in GD.^104^

We believe that, in general, GD per se is not sufficient reason for the use of systematic treatment with bisphosphonates, but informed use of bisphosphonates should not be excluded in patients with GD for appropriate treatment of any relevant condition. The effects and recommended administration of new bone agents (eg, denosumab) have not been established in GD.

### Areas of controversy OR no consensus

The effect of specific therapy on the evolution of the Erlenmeyer flask deformity is worthy of further study to help elucidate the pathogenesis of abnormal bone development in growing children with GD (see Fig. 1B).

In addition, further work is required to understand the implications for GD of blood concentrations of vitamin D and its metabolites.

### Remaining unmet needs

Several publications report improvement in BMD as a result of ERT or SRT.^18^, ^63^, ^105^

In children and adolescents, several studies have shown apparent bone mass accretion upon institution of enzyme therapy, with patients crossing centile lines for bone mass.^52^, ^106^ However, the clinical significance of these findings is not always clear, and methodological issues (eg, use of retrospective analyses, technical issues) and the absence of comparative data may be sources of bias. In children, there is an additional confounder in that delayed pubertal development and delayed growth are often ameliorated with the use of enzyme therapy, so that it is not clear whether any effect on BMD is direct or related to changes in hormonal status and bone growth.^72^ We are concerned that the poor predictive value of DXA for clinical outcomes is often ignored. Until a frank evaluation of the predictive power of DXA for fractures in GD has been performed, the use of DXA to predict the risk of fracture in GD patients is limited. The need remains for better strategies for determining bone integrity and fracture risk that are clinically useful, safe and convenient, transferable, robust, and valid.

## Osteonecrosis

### Natural history

Osteonecrosis, reported in up to 43% of adult GD patients in a UK cohort,^84^ can be seen as another consequence of the infiltration of the BM by Gaucher cells.^18^ New lesions can occur at any time throughout life. Osteonecrosis is often multifocal and may be associated with puberty or pregnancy.^107^ It significantly impairs quality of life for GD patients, commonly causing chronic pain and disability and leading to the need for surgical intervention.^108^

### Definition of terms

#### Osteonecrosis

The terms osteonecrosis, avascular necrosis, aseptic osteonecrosis, and bone infarct are used variably in the GD literature. They all describe the same clinical condition, though in some cases they depend on the anatomical location of the lesion. The etiology of this condition in GD is not clear, and any assumption of arterial insufficiency is not warranted; therefore, we prefer the term osteonecrosis.

This condition develops over weeks to months and has characteristic features on MRI that also evolve over time (Fig. 2). We contend that the location of the lesion is irrelevant to the terminology, although the location does influence the risk of late complications because involvement of the articular surface can lead to subchondral or joint collapse.

#### Bone crisis and infectious osteomyelitis

Osteonecrosis is often predicted by the occurrence of a bone crisis.^11^ This clinical event takes the form of acute, severe bone or joint pain that is often associated with fever, edema, and a local and systemic inflammatory response. The symptoms are often immobilizing, requiring bed rest and opiate analgesia. Clinically, bone crises can be confused with infectious osteomyelitis.^109^ In a bone crisis, no bacteremia is present.^15^, ^110^ Generally used imaging methods such as MRI, but also radionuclide bone and BM scintigraphy, can give information to separate a bone crisis from infectious osteomyelitis at an early clinical time point (1 to 3 days after pain onset). Radionuclide bone scans are usually initially “cold” in Gaucher bone crises, but “hot” in the presence of osteomyelitis. Detailed information about radionuclide imaging in GD is summarized in an overview by Mikosch et al.^111^ In clinical terms, it is important to note that bone crises are much more frequent than infectious osteomyelitis in GD.

#### Pseudo‐osteomyelitis

This term is confusing and must be eliminated from our vocabulary: it is a bone crisis.

### Pathogenesis

The mechanism of osteonecrosis is less clear in GD than in other disorders such as sickle cell disease and diving decompression injury in which arterial obstruction is proposed.^14^ The Gaucher cells, engorged with glucocerebroside, cause chronic inflammation with the production of several cytokines that have key influences on the bone remodeling process.^40^ *GBA1* genotype also has an influence, and p.Asn409Ser (formerly known as p.N370S) homozygosity appears to confer some protection against the early development of bone disease,^40^, ^42^, ^67^, ^112^ though associated with progressive skeletal disease of adult onset.^44^, ^113^

#### D‐dimer

D‐dimer concentrations vary significantly between patients, depending on the presence or absence of osteonecrosis, splenectomy, and treatment.^114^ Significantly higher concentrations of D‐dimer have been observed in GD patients than in a control group and in GD patients with abnormal MRI findings in the long bones than in those with normal long‐bone MRI findings.^115^ Presumably, these D‐dimer increases (along with reported increases in thrombin–antithrombin complexes in patients with osteonecrosis^116^) reflect a state of coagulation activation, but whether they are contributory or consequent to osteonecrosis remains unresolved.

Osteonecrosis is seen relatively frequently (46%) in patients with radiologically defined type B morphology (heterogeneous BM infiltration) in comparison with only 3% in patients with type A morphology.^24^, ^117^

#### Splenectomy

Splenectomy is an important risk factor for osteonecrosis before and even after initiation of ERT.^35^, ^84^, ^118^ After removal of the spleen, an overload of Gaucher cells occurs within the BM. It is likely that the development of osteonecrosis after splenectomy is facilitated by an alteration in thrombosis, thrombolysis, platelet activation, and rheological parameters (eg, blood viscosity, red cell deformability)^84^, ^119^, ^120^ and an increase in the number of microparticles that are normally cleared by the spleen.^121^ Splenectomized patients have a greater chronic inflammatory burden and more severe bone disease than nonsplenectomized patients.^67^ It has long been debated whether the association between osteonecrosis and splenectomy is causal or related to the greater disease burden in patients requiring splenectomy. The close temporal association between splenectomy and subsequent osteonecrosis events^84^ suggests a causal relation.

Therefore, splenectomy should be avoided to avert the development of osteonecrosis.^40^ On the other hand, splenectomy does not appear to be causally linked to osteopenia, further underlining the distinct pathophysiological mechanism involved.^122^ However, bone damage resulting from osteonecrosis is a risk factor for pathological fracture in GD patients.^110^

### Therapy

Delay in initiating specific treatment is also relevant to the development of osteonecrosis.^123^ Patients who start ERT within 2 years of diagnosis have a significantly lower risk of osteonecrosis than do those who receive treatment 2 years or more after diagnosis.^118^

### Our recommendations

#### Monitoring and scoring

We recommend plain radiography as part of the baseline assessment at diagnosis, for specific lesions, and as needed to interpret DXA scans. Plain radiography should be performed in anatomical areas where patients are symptomatic. However, plain radiography can detect only severe osteonecrosis with secondary and tertiary changes; therefore, it is not valid for an early diagnosis of osteonecrosis. MRI and scintigraphy are valid methods for monitoring acute bone crises. Only with time does it become clear whether an ongoing process in the bone is osteonecrosis rather than reversible marrow edema: Damage remaining after several years can be considered proof of osteonecrosis.

MRI, where available, should be repeated everyone to 2 years and when indicated for clinical reasons (pain, trauma, surveillance because of a history of osteonecrosis or splenectomy, and in patients with high DS3 scores), whereas the interval between two MRI scans may be increased in stable patients treated for more than 5 years.^55^ Expert guidance is needed for the use of MRI in children.

#### Treatment

Osteonecrosis is always an indication for GD‐specific therapy (ERT or SRT, even if no other criteria are fulfilled).

When a bone crisis occurs and MRI shows bone edema and periosteal infiltration on STIR or T2‐fat‐saturated sequences, bed rest to reduce the articular load can help to prevent progression and to relieve symptoms. In addition, appropriate analgesics, up to and including opioids, should be prescribed on the basis of pain severity.

Historically, surgical therapy for fractures or joint deformity has carried a risk of infections, particularly osteomyelitis, often related to the spread of cutaneous staphylococcal infection from indwelling cannula sites. Currently—and in experienced centers—joint replacement in GD is safe, and prostheses can function well for many years. Attention to risk of bleeding and infection is crucial. Operative bleeding caused by coagulation disorders and platelet dysfunction occur particularly in patients with hepatic involvement and in those who have undergone splenectomy. We advise extreme caution in the use of invasive procedures in the context of acute osteonecrosis in GD to reduce the risk of subsequent bone infection. We suggest that it is safest to presume that an acute onset of bone pain is related to osteonecrosis and to withhold emergent invasive intervention such as decompression or drainage, unless there is compelling evidence otherwise. We recommend the use of antibiotics when invasive procedures are unavoidable. Core decompression is not proven to be effective; therefore, we do not recommend it. Joint replacement may be required for the relief of chronic pain.

### Remaining unmet needs

Risk factors such as anemia,^10^ iron metabolism, cytokine expression, and genotype have not been conclusively correlated with osteonecrosis and should be investigated further.

Further investigation is needed to determine whether disease recurrence or progression on enzyme therapy depends on the ERT dose or occurs as a result of “treatment resistance.” If treatment resistance does occur, a biomarker is needed to predict its development. We should also consider the possibility of poor tissue penetration to local areas and disease “sanctuary sites” such as bone. It remains to be seen whether small molecule treatments will be more effective than ERT for preventing the recurrence of osteonecrosis.

## Conclusions

- Recommendations for monitoring presented here are based on an understanding and interpretation of the pathology as well as a retrospective review of the utility of the modalities.
- Bone disease in patients with GD is prevalent, disabling, and heterogeneous in pathology and clinical manifestations, in both the individual and the community.
- Evidence suggests that although the pathology of BM disease and mineral compartment are etiologically linked, separate consideration should be given to monitoring by MRI and DXA. Both yield useful information on the current state of disease and the likelihood of future events such as infarction, fractures, etc.
- Therapy should address GD‐specific challenges to bone, as well as conventional ones, and include, as for all bone diseases, generally appropriate attention to dietary (calcium and vitamin D) and lifestyle factors (suitable weight‐bearing exercises and avoidance of smoking and excess alcohol consumption).
- BM disease is rapidly responsive to GD‐specific therapy. Further work is required to understand the relation between an improvement in the bone disease score and a change in risk of bone events. Reduced BMD is associated with increased fracture risk, and we need to understand whether improvement translates into clinical effect.
- Careful and agreed definitions of terminology are needed to guide the interpretation of existing evidence and to guide the planning of future studies.

## Disclosures

DH has received honoraria for speaking and advisory boards and research support from Genzyme Sanofi and Shire. PM has received fees from Sanofi Genzyme, Shire, and Actelion for lectures, participation at meetings, and advisory boards. NB has received fees from Sanofi Genzyme and Shire for lectures, travel reimbursement, and participation on advisory boards. FC has received fees from Sanofi Genzyme and Shire for lectures, participation at meetings and advisory boards, and travel reimbursement. TMC has received fees from Sanofi Genzyme, Shire, Actelion, and Amicus for lectures, travel reimbursement, and participation on advisory boards. OGA has received honoraria and travel reimbursement from Sanofi Genzyme. AK has received fees from Sanofi Genzyme, Shire, Amgen, and MEDA for lectures, travel reimbursement, and participation on advisory boards. PKM is a recipient of research grant, lecture fee honoraria, and travel support from Sanofi Genzyme and is a consultant for Sanofi Genzyme. LP has nothing to disclose. NW has received research grant and honoraria for participating in CME programs from Sanofi Genzyme, and honoraria and travel reimbursement for participation in medical advisory boards from Sanofi Genzyme, Shire HGT, and Pfizer. PD has received honoraria for speaking and advisory boards from Sanofi Genzyme and Shire and has received research support from Sanofi Genzyme.
